# Solid phase wax coating of *N*-acetylcysteine (NAC) to decrease its solubility profile as a ready to mix supplement†

**DOI:** 10.1039/d1ra09279k

**Published:** 2022-06-14

**Authors:** Sara Madarshahian, Mojtaba Enayati, Gerard Vinyes Parés, Gerhard Ufheil, Alireza Abbaspourrad

**Affiliations:** Department of Food Science, College of Agriculture and Life Sciences, Cornell University Ithaca 14853 NY USA alireza@cornell.edu; Nestlé Product Technology Center Nestlé Health Science Bridgewater NJ 08807 USA; Nestlé Research Lausanne 26 Switzerland

## Abstract

*N*-Acetylcysteine (NAC) has health benefits attributed to its antioxidant properties and disulfide bond cleavage ability. Unfortunately, solutions of NAC are acidic with an undesirable taste and an unpleasant aftertaste. A method for slowing NAC release in water was developed using a solid phase wax coating. A coating of natural waxes, using food grade corn oil as the solvent and surfactants to facilitate the wax coating on the particles was used to decrease the solubility of NAC powder, crystals, and granules in water. A high NAC loading, between 55 and 91% for NAC granules and NAC crystals, was achieved as measured using LC-MS. The NAC wax-coated particles were fully characterized, and microscopy and SEM images revealed the shape, morphology, and size of the particles. Conductometry was used to study NAC release profile in water from wax-coated particles and the results indicate that solid phase wax coatings slowed the release of NAC into water.

## Abstract 2

*N*-Acetylcysteine (NAC) has significant health benefits. Wax coating is applied to granules, crystals, and powdered NAC to make it more palatable by delaying its release in water.

## Graphical abstract

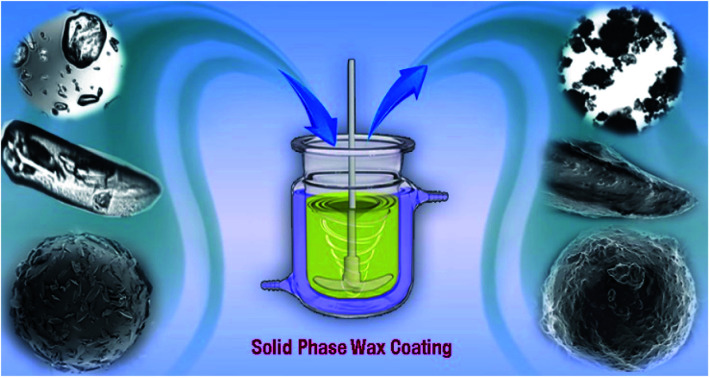

## 1 Introduction

People are more health conscious today and the food industry is responding by finding novel ways of including nutritional supplements such *N*-acetylcysteine (NAC) to enhance their products. NAC has been used for a wide variety of medical applications from respiratory diseases to cancer therapy, acetaminophen toxicity and psychiatric disorders.^1,2^ Its primary mode of action is through antioxidant and anti-inflammatory activity.^3^ Further, oral deliver is the safest and preferred delivery method.^4,5^

Unfortunately, despite NAC's wide range of medical benefits, it is very astringent and smells unpleasantly of sulfur, making oral delivery by solution difficult especially for high-dose NAC therapy.^2^ To combat these problems in similar situations food chemists have developed coatings for supplements that mask unpleasant flavors while also protecting against unwanted reactions and product degradation.^6–8^ These coatings can increase the shelf-life of sensitive ingredients and control their release profiles under certain conditions. Ideally, coating materials should not react with the core materials while at the same time they should seal, protect and release the core under specific conditions.^9,10^

The US Food and Drug Administration (FDA) and the European Union (E901-903) call for the use of natural waxes in the food and pharmaceutical industry for coatings of water-soluble materials because of their minimal impact on the coated materials and their inherent hydrophobicity.^11–14^ Beeswax (BW), rice bran wax (RBW), candelilla wax (CanW), and carnauba wax (CW) are examples of food-grade natural waxes currently used to coat different food and drug compounds.^15,16^ Carnauba wax has the highest melting point and has been used to encapsulate a wide variety of hydrophilic materials^17^ such as valproic acid,^18^ ketoprofen,^19^ ascorbic acid,^20^ and drugs such as captopril and metformin hydrochloride.^21^ Carnauba wax and beeswax were used for encapsulation of water-soluble colorants such as toluidine blue dye.^14^ Beeswax has been used to encapsulate fluorouracil and ftorafur,^22^ rice bran wax has been used in food products,^23^ and candelilla wax has been used for phosphate fertilizer encapsulation.^24^

The goal, therefore, is to create a system to deliver high NAC dosage by encapsulating NAC in a food safe manner, that masks the undesirable mouth feel of NAC solutions using coatings or encapsulation. Previously, NAC was encapsulated by poly(lactic-*co*-glycolic acid) (PLGA) and inside mesoporous silica geometries such as hexagonal MCM-41 (Mobil Composition of Matter No. 41) and cubic MCM-48 (Mobil Composition of Matter No. 48).^25^ However, MCM-41 and MCM-48 are not acceptable at the narrow range of food-grade materials. While there are also reports of liposomal encapsulation of NAC using different phospholipids all use toxic organic solvents such as such as chloroform.^26,27^ Further, all of these systems only offer low NAC loading for a very specific use not as a general nutritional supplement.

We have developed a method for solid phase wax coating of NAC using natural food safe waxes, non-toxic organic solvents, and corn oil as the dispersion solvent. NAC in three forms, powder, crystals, and spherical granules (prepared *via* suspension granulation)^28^ were used to prepare high loading products containing more than 55 wt% NAC. A uniform wax coating was confirmed *via* morphological studies using scanning electron microscopy (SEM) and controlled release of NAC was confirmed using conductometry in aqueous solutions.

## 2 Experimental section

### 2.1 Materials

NAC crystals and NAC powder (80 mesh) were used as provided by Nestle. Candelilla wax (CanW, melting point 68–72 °C), carnauba wax (CW, melting point 82 °C, no. 1 yellow, refined), sorbitan monooleate or Span 80 (saponification value: 145 to 160), formic acid (FA 98%) and acetonitrile (HPLC grade, ≥99.9%) were purchased from Sigma-Aldrich. Rice bran wax (RBW, melting point 79–85 °C) was purchased from Nutley's Kitchen Gardens. Beeswax (BW, DR-101, melting point 62–65 °C) was purchased from Strahl & Pitsch, Inc. Xanthan gum was purchased from TIC Gums. Carboxymethyl cellulose (CMC) was purchased from Sigma (low viscosity, 50–200 mPa s, 4% in water). Dioctyl sulfosuccinate sodium salt or AOT 96% was obtained from VWR. Hydroxypropyl methylcellulose (HPMC) purchased from Fisher (40–60 mPa s, 2% in water), sorbitan monostearate or Span 60 purchased from TCI America (saponification value: 145 to 156), and hexanes were purchased from Fisher (≥98.5%, Certified ACS, Fisher). Corn oil was purchased from a local market. Milli-Q and DI water were used for analyses and experiments, respectively. All chemicals were used as received without any further purification.

### 2.2 General procedure for NAC solid-phase wax coating

NAC powder and crystals were coated directly as received and NAC granules were produced as described below and coated using the same method as described for powder and crystals.

#### 2.2.1 Preparation of NAC granules

NAC granules were prepared as reported.^28^ In brief, we used a concentrated and viscous solution of NAC, gum and surfactant (Table 3) that was added to warm corn oil with stirring and heating, then the NAC granules were allowed to precipitate with slow cooling. The granules were washed with cold hexane to remove extra oil and then dried under vacuum.

#### 2.2.2 NAC particle coating

**Scheme 1:**
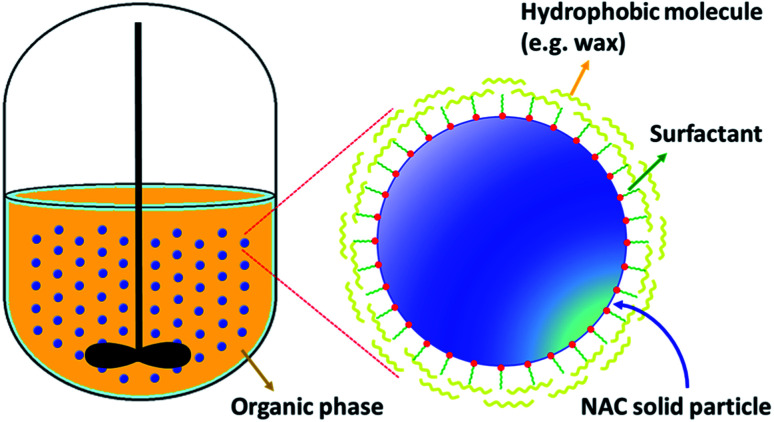
Schematic of solid-phase NAC wax coating process.

The general procedure for coating all types of NAC particles with the various waxes was the same, Tables 1–3 contains the amounts of NAC, wax and surfactants used for each sample. The oil serves as the solvent and we present here the procedure for coating NAC powder with carnauba wax (CW) (sample P6-CW) as an example. Corn oil (100 mL) was added to a jacketed glass reactor, equipped with a mechanical stirrer and a temperature-controlled circulating water bath, and heated at 70 to 80 °C. Carnauba wax (1.5 g) was added to the heated oil and after the wax was completely dissolved, a surfactant (0.5 g, 5 wt% based on NAC) was added and allowed to stir for 15–20 minutes. NAC powder (8.0 g) was then added to the reactor and stirring continued at 400 rpm. To prevent the wax coated NAC from precipitating too quickly and forming an intractable solid mass on the bottom of the flask, the temperature of the reaction was decreased at an approximate ramp of 5 °C every 30 min to reach a final temperature of 25 °C. After the reaction reached room temperature, it was stirred for an additional hour. Then stirring was stopped and the wax coated NAC settled to the bottom of the flask. The extra corn oil was decanted, and the wax coated NAC was washed with cold hexane and vacuum filtered to dry. Scheme 1 represents the process of solid-phase NAC wax coating. All other sample compositions are represented in Tables 1–3 and were prepared in a similar manner.

**Table 1:** Reaction conditions for coating NAC powder with hydrophobic natural waxes

Sample code	Composition of NAC coating reactions			Theo/exp. NAC loading (%)	NAC release (%)^a^ in 5 min
	NAC (g)	Wax (g)	Surfactant (g)		
**NAC powder (P) coating with hydrophobic natural waxes**					
**P1**-RBW^b^	80 mesh (8.0 g)	RBW (1.5 g)	Span 60 (0.5 g)	80.0/76.9 ± 3.7	75.9
**P2**-CanW^c^	80 mesh (8.0 g)	CanW (1.5 g)	Span 60 (0.5 g)	80.0/73.2 ± 0.2	28.9
**P**-BW^d^^e^	80 mesh (7.5 g)	BW (2.0 g)	Span 60 (0.5 g)	75.0/68.5 ± 0.6	37.3
**P4**-BW	80 mesh (8.0 g)	BW (1.5 g)	Span 60 (0.5 g)	80.0/66.8 ± 0.8	25.3
**P5**-CW^f^	80 mesh (7.9)	CW (1.6 g)	Span 80 (0.5 g)	79.0/73.2 ± 1.3	81.2
**P6**-CW^g^	80 mesh (7.4)	CW (2.1 g)	Span 80 (0.5 g)	74.0/66.9 ± 6.9	77.2
**P7**-CW	80 mesh (8.0 g)	CW (1.5 g)	Span 60 (0.5 g)	80.0/81.1 ± 3.2	41.3
**P8**-CW^h^	Ground powder ≤ 125 μm (7.0 g)	CW (2.5 g)	Span 60 (0.5 g)	70.0/57.1 ± 0.5	8.3

a Percent reported is the percentage of 2.5 wt% NAC used.

b RBW: rice bran wax.

c CanW: candelilla wax.

d Homogenized at 70 °C for 5 min.

e BW: beeswax.

f Carnauba wax (CW).

g Higher molecular weight CW was used compared to P5.

h NAC crystals ground and sieved.

**Table 2:** Reaction conditions for coating NAC crystals with hydrophobic natural waxes

Sample code	Composition of NAC coating reactions			Theo/exp. NAC loading (%)	NAC release (%)^a^ in 5 min
	NAC (g)	Wax (g)	Surfactant (g)		
**NAC crystals (C) coating with hydrophobic natural waxes**					
**C1**-RBW^b^	Crystals 350–1000 μm (8.0 g)	RBW (1.5 g)	Span 80 (0.5 g)	80.0/81.1 ± 4.6	97.7
**C2**-CanW^c^	Crystals 350–1000 μm (8.0 g)	CanW (1.5 g)	Span 80 (0.5 g)	80.0/91.7 ± 2.6	78.9
**C3**-BW^d^	Crystals 350–1000 μm (8.0 g)	BW (1.5 g)	Span 80 (0.5 g)	80.0/86.2 ± 3.1	99.0
**C4**-CW^e^	Crystals 350–1000 μm (8.0 g)	CW (1.5 g)	Span 80 (0.5 g)	80.0/64.0 ± 2.2	23.9
**C5**-CW	Crystals 350–1000 μm (8.0 g)	CW (1.5 g)	Span 60 (0.5 g)	80.0/75.9 ± 4.8	17.3
**C6**-CW	Crystals 350–1000 μm (8.0 g)	CW (1.5 g)	Span 80 (0.5 g)	80.0/90.1 ± 1.8	27.7
**C7**-CW^f^	Crystals 350–1000 μm (8.0 g)	CW (1.5 g)	Span 80 (0.5 g)	80.0/83.7 ± 0.0	5.5

a Percent reported is the percentage of 2.5 wt% NAC used.

b RBW: rice bran wax.

c CanW: candelilla wax.

d BW: beeswax.

e Carnauba wax (CW).

f Not washed with hexane, bulk corn oil, removed by filtration.

**Table 3:** Reaction conditions for coating NAC granules with hydrophobic natural waxes

Sample code	Composition of NAC oating reactions			Theo/exp. NAC loading (%)	NAC release (%)^a^ in 5 min
	NAC (g)	Wax (g)	Surfactant (g)		
**Coating of NAC granules (G) (prepared as indicated below) with hydrophobic natural waxes**					
**G1**-BW^b^	NAC granules (8.0 g) [NAC + HPMC + AOT]: [80 : 15 : 5]	BW (1.5 g)	Span 60 (0.5 g)	65.0/55.3 ± 7.1	65.7
**G2**-BW	NAC granules (7.5 g) [NAC + CMC + AOT]: [80 : 15 : 5]	BW (2.0 g)	Span 80 (0.5 g)	60/88.1 ± 2.2	57.0
**G3**-CW^c^	NAC granules (8.0 g) [NAC + XG + Span 60]: [93 : 2 : 5]	CW (1.5 g)	Span 60 (0.5 g)	74.4/86.3 ± 0.0	66.3
**G4**-CW	NAC granules (8.0 g) [NAC + HPMC + AOT]: [80 : 15 : 5]	CW (1.5 g)	Span 60 (0.5 g)	65.0/56.4 ± 3.7	34.8
**G5**-CW	NAC granules (7.5 g) [NAC + CMC + AOT]: [75 : 20 : 5]	CW (2.0 g)	Span 60 (0.5 g)	57.0/54.7 ± 3.3	32.2
**G6**-CW	NAC granules (7.5 g) [NAC + HPMC + AOT]: [75 : 20 : 5]	CW (2.0 g)	Span 60 (0.5 g)	57.0/56.9 ± 2.3	17.0

a Percent reported is the percentage of 2.5 wt% NAC used.

b BW: beeswax.

c Carnauba wax (CW).

### 2.3 Characterization

Wax coated NAC particles were characterized by LCMS and conductometry to access loading capacity and release profile, FTIR was used to confirm the presence of NAC and microscopy was used to investigate surface morphology.

#### 2.3.1 Assessment of NAC loading

To assess the efficiency of the wax coating of the different forms of NAC, samples of the wax coated NAC was analyzed using LCMS. Samples for analysis were prepared by grinding the samples to a fine powder, then 7.5 mg of the powder was placed into 20 mL vials containing 15 mL 0.1% v/v formic acid in fresh Milli-Q water. All samples were shaken on an Orbital Shaker to extract the NAC from the wax. After 24 hours the samples were filtered and used for LC-MS analysis. The theoretical loading percentage *versus* the experimental NAC loading percentage are reported in Tables 1–3. All samples were analyzed in triplicate and the calibration curve used can be found in the Fig. S2.†

#### 2.3.2 LCMS analysis

A method to determine NAC loading was developed for LCMS. Our LC (Agilent 1100 series) is equipped with a mass spectrometer and a Luna Omega LC column (Phenomenex, 100 × 4.6 mm, 3 μm, Polar C18 100 Å) was used for reverse-phase chromatography. LC eluents were solution A: DI-water (formic acid 0.1%) and solution B: acetonitrile under gradient elution (Table S1, ESI†). The flow rate was 0.3 mL min^−1^ and injection volume was 10 μL. The column temperature was maintained at room temperature and the overall run time of each sample was 12 minutes. The mass spectrometer (Finnigan LTQ mass spectrometer) equipped with an electrospray interface (ESI) in positive electrospray ionization mode was used for the mass spectral data acquisition of NAC. The optimized parameters are sheath gas flow rate at 20 arbitrary units, spray voltage set at 4.00 kV, the capillary temperature at 350 °C, capillary voltage at 41.0 V, and tube lens voltage set at 125.0 V. The NAC calibration curve is provided in ESI (Fig. S2†).

#### 2.3.3 ATR-FTIR spectroscopy analysis

We used a Shimadzu IRAffinity-1S FTIR spectrophotometer equipped with attenuated total reflectance (ATR) to analyze the wax coated granules. Samples were scanned 64 times in the selected spectral range from 400 to 4000 cm^−1^ with the resolution of 2 cm^−1^. The data was analyzed by LabSolution IR software.

#### 2.3.4 Microscopy and SEM image acquisition

The visualization of NAC particles was done by LEICA DFC 3000 G bright field microscope. Samples were placed on the microscope slide and a 50 : 50 solution of glycerol and water was added to wet the samples and provide the contrast for core–shell identification. Photos of samples were taken before and after wetting. To observe the morphology of NAC coated particles, scanning electron microscopy (SEM) micrographs were taken for selected coated samples from NAC powder, NAC crystals, and NAC granules. A Zeiss Gemini 500 Field Emission SEM was used to do this. Before being mounted on the SEM machine, all of the samples were coated with an ultra-thin gold layer using a sputter coater.

### 2.4 NAC release profile in water by conductometry

Conductivity was measured using Metrohm 856 Conductometer, equipped with a 5-ring probe electrode (0.7 constant, conductivity range of 5–20 mS cm^−1^). A 100 mL beaker (OD: 5 cm, ID: 4.5 cm, 7.5 cm) was chosen for NAC release, any change to the reaction vessel or stir bar size resulted in different conductivity results. Samples were added in one addition to DI water that had been stirring at 500 RPM using a magnetic stirring bar (2.0× 0.7 cm) for 60 s at ambient temperature. For all samples, a specific amount is calculated in order to provide 2.5 wt% of NAC in water if all NAC is released, providing a high dosage (2500 mg of NAC) in 100 mL of water. The conductometric information was recorded using the software Tiamo, Version 2.5 for 600 s or more in few cases. In addition, a calibration curve of conductivity *versus* concentration for pure NAC was used to estimate NAC concentration release from wax coated NAC samples (Fig. S1†). All samples were measured twice to ensure that the curves were similar and a third party also measured the conductivity of the samples and achieved similar results.

### 2.5 Statistical methods

All LC-MS measurements were carried out in triplicate and Microsoft Excel was used to calculate the standard deviations and perform the Student's *t*-test (*p* < 0.05).

## 3 Results and discussion

NAC is a small molecule with high water solubility (20 wt% at room temperature). With many health advantages, however, using pure NAC directly in water for drinking is unpalatable due to its high acidity, unpleasant smell, and the sour and bitter taste, including an undesirable aftertaste. We proposed that coating or encapsulating NAC would be an effective way to decrease the solubility of NAC in water. To do this, we used solid-phase wax coating methods and food safe naturally available waxes to coat NAC powder 80 mesh (P), NAC crystals, 350–1000 μm (C), and NAC granules using suspension experiments (G). The compositions and reaction conditions of these three different NAC solid phases that were wax coated are presented in Tables 1–3. One of the major benefits of the NAC solid phase coating, crystal and powder, is that there is no need for preparation of NAC solutions and therefore, no extra time and energy is needed for removing water to form the particles.

The waxes that we used in this study, are only soluble in oil at high temperatures. As the solution cools, the wax will begin to solidify and separate from the oil and the solution will slowly form two phases upon cooling. This means that the main driving force would be the crystallization of wax at lower temperatures. Therefore, by using an appropriate surfactant in the oil/wax/NAC solution that can act like a compatibilizer, and increase adhesion between the hydrophilic NAC and hydrophobic wax, we observed that the wax deposited on the NAC particle surface. The lipophilic head of the surfactant attracts and adheres to the wax, while the hydrophilic head adheres to the NAC. In this way, the lipophilic head of the surfactant serves as a nucleation site, and attractant, for the wax molecules as they solidify with dropping solution temperatures, thus, they gradually coat the NAC molecule. This was confirmed by our microscopy images and also by the NAC release data.

### 3.1 NAC release profile of the NAC wax-coated samples by conductometry

**Fig. 1:**
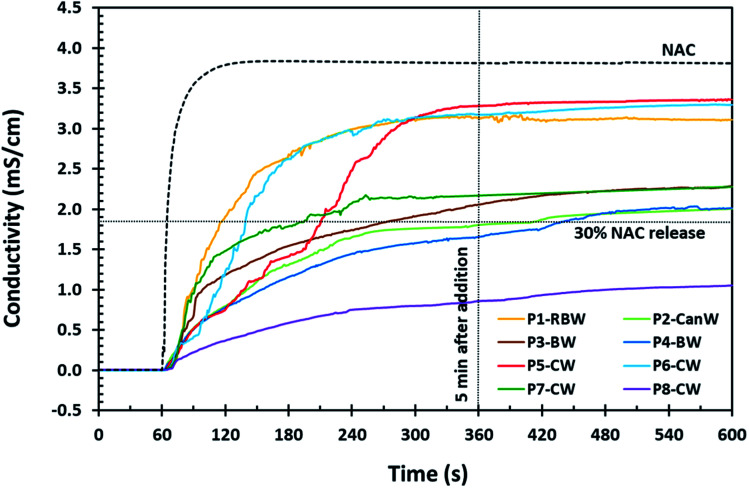
NAC release profile of the 80 mesh powder (P) samples coated with natural waxes by conductometry. Reaction conditions for these samples are presented in Table 1.

**Fig. 2:**
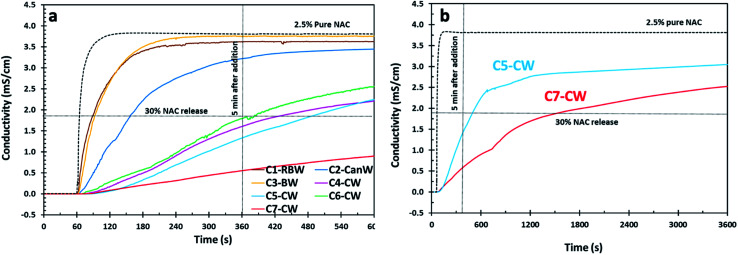
(a) NAC release profile for the NAC crystal (C) samples coated with natural waxes by conductometry. Reaction conditions for these samples are presented in Table 2. (b) Release profile of C5-CW and C7-CW for 60 min.

**Fig. 3:**
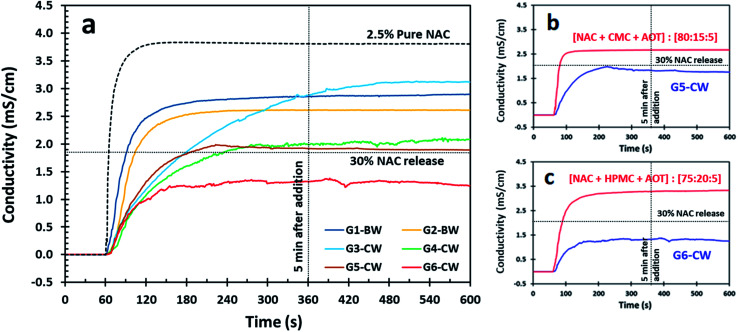
(a) Release profile for the NAC granules by conductometry; granules (G) have prepared previously *via* suspension experiment, and then wax coating with natural waxes. (b and c) Comparison of release profile of granules and G5-CW and G6-CW by conductometry, respectively. The red line is the release profile of the granules from the suspension experiment and the blue line is the release profile from the particles after wax coating samples G5-CW and G6-CW.

NAC is acidic in water and as such, conductometry has been shown to be a useful and reproducible method to estimate the concentration of NAC released through dissolution.^28,29^ Conductometry provides a fast, real-time method for determining the concentration of NAC in solution because the concentration of NAC in solution and the solution conductivity are proportional. Using conductometry means that the samples do not have to be quenched to assess the concentration and we can monitor the dissolution directly. Therefore, we used conductivity to measure the release of NAC from our coated samples into DI water using a calibration curve. The conductivity profile of 2.5 wt% of pure NAC is presented for comparison with the NAC release from wax-coated products (Fig. 1–3 and S3†). We chose 2.5 wt% of NAC as some conditions for which NAC has been shown to be effective, such as cystic fibrosis and chronic obstructive pulmonary disease (COPD), require high dosages of NAC (2000–6000 mg) therefore 2.5 wt% in 100 mL of water would provide a dose of 2500 mg.^2^ Further, we chose 5 minutes as an acceptable time for the material to be dissolved and then consumed while a 30% NAC release would then mean that approximately 600 mg would be dissolved which was found to be palatable. Thus we graphed our data with intersection lines of 30% NAC release and 5 min after addition.

Based on the NAC release profiles for NAC powder (Fig. 1), carnauba wax (CW) gives the lowest NAC release which corresponds to the highest coating efficiency *via* solid-phase wax-coating. Beeswax (BW) and candelilla wax (CanW) in P4-BW and P2-CanW, respectively, demonstrate acceptable results as well, remaining under 30 wt% NAC for 7 minutes.

NAC crystals used for wax coating experiments (Fig. 2a), were filtered to be in the range of 350–1000 μm size using two successive metal sieves, the first discarding larger than 1000 μm and the second for eliminating particles smaller than 350 μm. In the case of C7-CW, the sample was not washed with hexanes, the corn oil was removed only by vacuum filtration, and as a result it shows a noticeably low release of NAC in comparison with C5-CW which has the same composition (Fig. 2a). We concluded that washing with hexanes results in increased NAC release, most likely because it can create cracks in the coating materials on NAC crystals. This theory was confirmed by SEM.

Because of their notably slow release in the first five minutes, the release profile of C5-CW and C7-CW was studied *via* conductometry for 60 min (Fig. 2b). The conductivity of samples C5-CW and C7-CW increase gradually with time indicating that NAC releases gradually from these formulations.

NAC granules were prepared in a suspension experiment without hydrophobic coating and were then coated in a hydrophobic wax coating. Advantages of coating NAC granules is that these particles are spherical and can be made different sizes as needed.^28,30^ The disadvantages of NAC granules include their preparation, which requires two steps involving the dissolution in water and the subsequent removal of water, and NAC loading which is typically lower in granule formulations (Table 3) as gums are used as structuring for the granulation step. The release profile for G6-CW showed a slow release in the first five minute below the threshold of 30 wt% NAC (Fig. 3). G4-CW and G5-CW were also good, remaining at or only slightly above 30 wt% NAC but the initial release for all NAC granules was faster than the NAC powder and crystals.

The conductivity profiles of NAC for samples G5-CW and G6-CW wax coated samples were compared to the uncoated granules after preparation (Fig. 3b and c). Although the mass of the samples were normalized to have the same mass of NAC, both wax coated samples (G5-CW and G6-CW) showed a decrease in NAC release. Sample G6-CW, which contains 20 wt% hydroxypropyl methylcellulose (HPMC), indicates a balance between the gum and the CW coating exhibited and extended-release profile.

### 3.2 Structural identification of NAC coated wax particles by ATR-FTIR

To study the structure of the NAC wax-coating particles, the ATR-FTIR spectra of the starting materials and products were prepared and compared (Fig. S4 and S5†). The ATR-FTIR spectra of the products show the distinct bands at 3370 and 2550 cm^−1^ related to the N–H and S–H bonds, respectively, which proved the presence of NAC in the particles. Moreover, bands at 2915 and 2850 cm^−1^ are related to the C–H (aliphatic) bond of the natural waxes.^31^ All samples have a broad band with a small shoulder in the carbonyl region that can be attributed to both the NAC and natural wax. These results confirmed the presence of the NAC in the products which is further confirmed by LC-MS data (next section). Also, samples of wax coated NAC powder, crystal and granule were exposed to water for ten minutes after which time, the solution was vacuum filtered and the residual solid was air dried and analyzed using ATR-FTIR (Fig. S6†). The results of this analysis which shows the absence of NAC (seen by the 2550 cm^−1^ absorption band of –SH) for P5-CW and some residual NAC inside the samples for C2-CanW and G6-CW samples even after 10 min stirring in water. These results also confirmed by the conductometry results in which samples that took the longest time to release the NAC retained some NAC in the residual solids.

### 3.3 Determination of NAC loading by LC-MS

The sample preparation was designed to theoretically achieve 60 wt% or higher of NAC loading which was achieved for all but G5-CW and G6-CW which were approximately 57 wt% (Table 3). To determine the actual NAC loading in the products, we used the method described above. A Selected Reaction Monitoring (SRM) chromatogram and mass spectrum of pure NAC and C5-CW were presented in Fig. S7 (ESI†). The experimental NAC loading calculated based on measuring the peak area of each SRM, were reported in Tables 1–3. As can be seen in Tables 1–3, for majority of the samples, the theoretical and experimental NAC loading are in good agreement. However, for some of the samples the experimental NAC loading is higher than theoretical value, which is likely due to not all of the wax that is dissolved in oil was used for the coating and a lower amount of the wax deposited on the samples.

For any particular sample, there is a distribution (range) of well coated NAC particles and poorly coated NAC particles. The poorly coated portion of the same sample can be a result of sticking and subsequent breaking of NAC particles, introducing cracks into some particles due to temperature changes and/or inappropriate handling, or inefficient coating of some of the NAC particles in the reaction mixture. The fast increase in the conductivity of some sample followed by a decreased release can be a direct result of releasing NAC from these imperfectly coated samples, and when the NAC in these samples is released, the conductivity trace becomes steady over time. However, NAC loading measured by LC-MS is obtained by grinding of samples to mechanically break and destroy the coating which allows for the direct measurement all of the NAC at once.

### 3.4 Morphology of the wax coated NAC particles

**Fig. 4:**
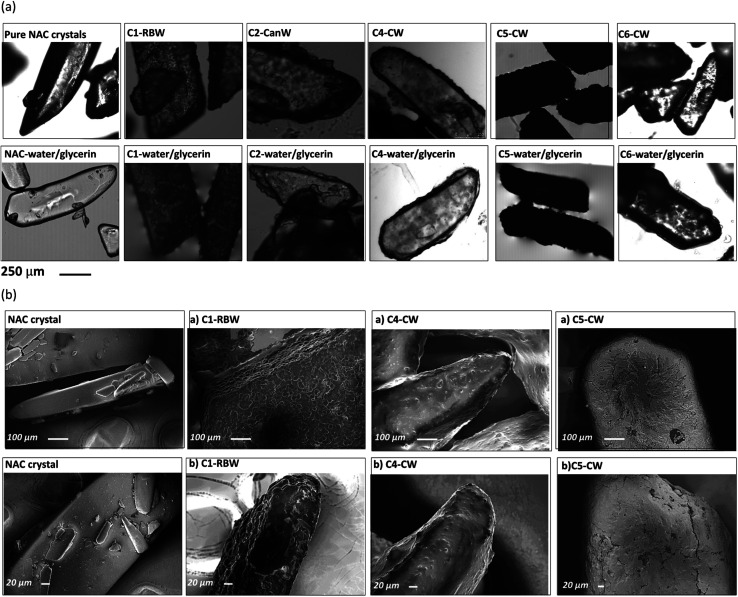
(a) Microscopic images of pure NAC crystals and NAC crystals coated with different waxes, (b) SEM images of pure NAC crystals and wax coated NAC crystals.

After coating the NAC crystals have an off-white coating on the crystal surfaces (Fig. S8†), despite this the particles are not sticky and do not agglomerate. Microscopy images of wax-coated NAC crystals before and after water/glycerin (50% v/v) addition confirmed that a film of wax formed on the surface of crystals upon coating (Fig. 4a). The shape and size of the NAC crystal is determined by and corresponds to the starting crystals. SEM of the NAC crystals and coated NAC crystals reveals the wax coating and the irregularity of the coating (Fig. 4b). For example, sample C5-CW clearly reveals cracks in the wax coating. Microscopy and SEM images of wax-coated NAC powder particles show the irregular and agglomerated particles due to the small size of the NAC powder (80 mesh) (Fig. S9 and S10†).

**Fig. 5:**
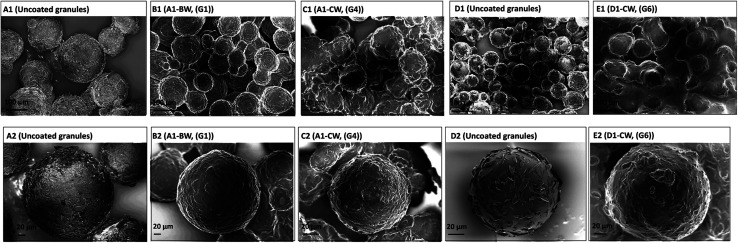
SEM images of the NAC granules (prepared *via* phase separation) coating with different waxes. Granules (A1 and A2) were made from NAC + HPMC + AOT: 80 : 15 : 5. Granules marked (D1 and D2) were made using NAC + HPMC + AOT: 75 : 20 : 5. (B1, B2 and C1, C2) are granules A1 coated with BW (G1) and CW (G4), respectively. (E1 and E2) are granule (D1 and D2) coated with CW (G6). The top row are bulk samples and bottom row are magnifications of a single particle from the top row images.

The SEM of the uncoated granulated particles reveals the presence of NAC crystals on the surface of the particles due to recrystallization before wax coating (Fig. 5). These NAC surface crystals are not present after the coating, showing the efficiency of the process (Fig. 5). In addition, it can be seen that these granulated particles are spherical and they can be produced in desired particle size.

The SEM images of the granulated samples illustrate their spherical nature and the core shell structure after wax coating is clearly observed (Fig. S11†). Upon exposure to water, water penetrates into the layer of the wax, gradually releasing the NAC.

The cooling rate is an important factor in the wax coating. Higher rates of cooling caused the wax to precipitate in the reactor rather than on the surface of NAC, while moderate cooling rates helped the surfactants arrange the hydrophilic heads toward the NAC particle and the lipophilic heads towards the wax facilitating the coating of the wax onto the NAC particles. Therefore, choosing the proper surfactant is crucial. The two surfactants used, Span 60 and Span 80, are very close in HLB values and chemical structure. One important difference, though, is the melting temperature as Span 80 is a liquid at room temperature while Span 60 is a solid with melting point of 53 °C. Since the main approach for the wax coating is to slowly cool the hot solution of wax so a stepwise precipitation/coating occur on the surface of the NAC particles, using Span 60 which is a solid at room temperature and can co-precipitate with the wax onto the NAC particle surface provided the best coating and as a result decreased the rate of NAC release.

## 4 Conclusion

NAC aqueous solutions suffer from sour/bitterness and an unpleasant aftertaste that lingers for hours. To overcome these shortcomings, we have used solid-phase wax coating to coat NAC so that it can be dispersed in aqueous solution for oral delivery. The wax coatings on the NAC powder and crystals provided a product with irregular shape, NAC granules had a spherical shape and controllable size, however, NAC granules should be synthesized and dried in a separate method before being used for the solid phase wax coating. Our SEM results showed that carnauba wax and Span 60 provides the most uniform coating. The wax coating method that presented here requires a simple formulation and setup, is an industrially viable approach, and can be performed on NAC powder, crystals, and granules. Since NAC dissolution is not required in the process of solid wax coating, there is no need for the tedious and energy consuming water removal to obtain a solid product. This temperature dependent solid phase coating method is a promising way to produce NAC products suitable for oral formulation.

## Conflicts of interest

The authors declare no competing financial interest.

## Supplementary Material

RA-012-D1RA09279K-s001
