# Supplementary material for: Solid phase wax coating of N-acetylcysteine (NAC) to decrease its solubility profile as a ready to mix supplement
Source: RSC Adv. 2022 Jun 14;12(27):17550–8. doi: 10.1039/d1ra09279k (PMC9192162; doi:10.1039/d1ra09279k)
Supplement: RA-012-D1RA09279K-s001 [file RA-012-D1RA09279K-s001.pdf]

Electronic Supplementary Material (ESI) for RSC Advances.
This journal is © The Royal Society of Chemistry 2022

Supporting Information

Solid phase wax coating of N-acetylcysteine (NAC) to decrease its solubility profile as ready to mix supplement

Sara Madarshahian[a]†, Mojtaba Enayati[a]†, Gerard Vinyes Parés[b], Gerhard Ufheil[c], and Alireza Abbaspourrad[a] *

[a]Department of Food Science, College of Agriculture and Life Sciences, Cornell University, Ithaca 14853, NY, USA

[b]Nestlé Product Technology Center, Nestlé Health Science, Bridgewater, NJ 08807, USA

[c]Nestlé Research, Lausanne 26, Switzerland

†These authors contributed equally to this work.

*Corresponding Author E-mail: alireza@cornell.edu (A.A).

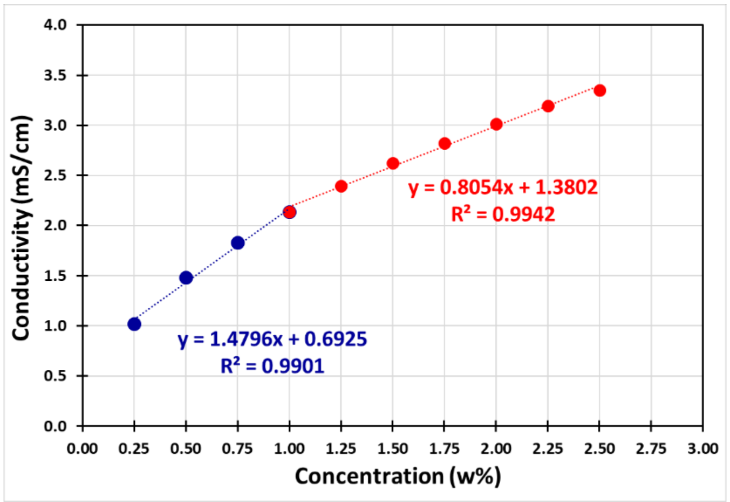

Figure S1. NAC calibration curve of conductivity vs. concentration in water.

Table S1. Gradient elution program of LC eluents.

Time (min)	Flow rate (ml/min)	H_2O (0.1% FA) %	ACN%
**0**	0.3	90	10
**1**	0.3	90	10
**5**	0.3	50	50
**8**	0.3	50	50
**11**	0.3	90	10
**12**	0.3	90	10

Preparation of stock solutions and calibration curves for LC-MS analysis

Stock solutions for standard solution were prepared by using 15 mg of NAC into 15 mL Milli-Q-water and FA 0.1% and vortexed at the ambient condition. The standard solutions were made in 10, 25, 50, and 100 ppm for low concentrations and also 100, 200, 300, 400, and 500 ppm for high concentrations. Both of calibration curves at low and high concentrations were linear (R^2 for series of low and high concentrations of NAC were 0.98 and 1.00, respectively, **Figure S2**). It was clear that there was a different detector response at concentration higher than 100 ppm and therefore two different calibration regions were used to determine concentration of NAC in the samples.

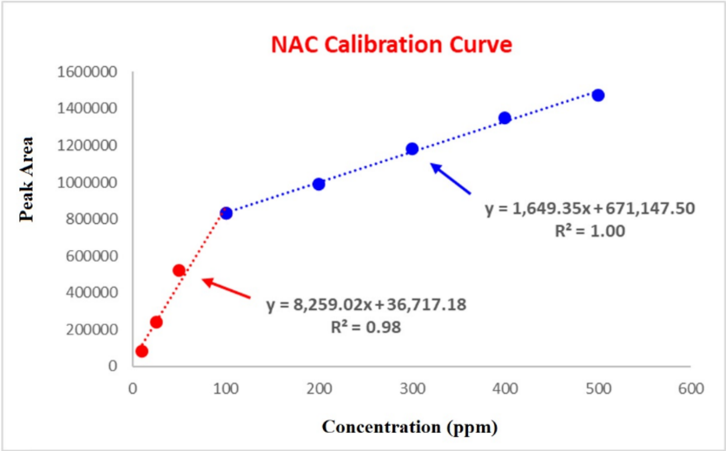

Figure S2. Calibration curve of pure NAC solutions (10-500 ppm) by LC-MS.

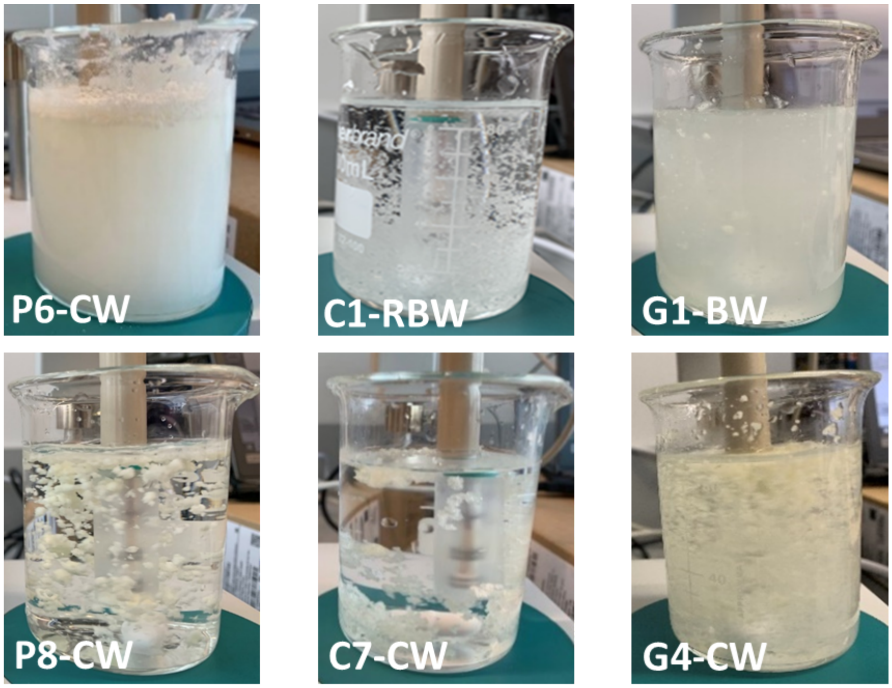

Figure S3. Images of representative samples during the conductometric measurements in water illustrated how the different samples disperse in water.

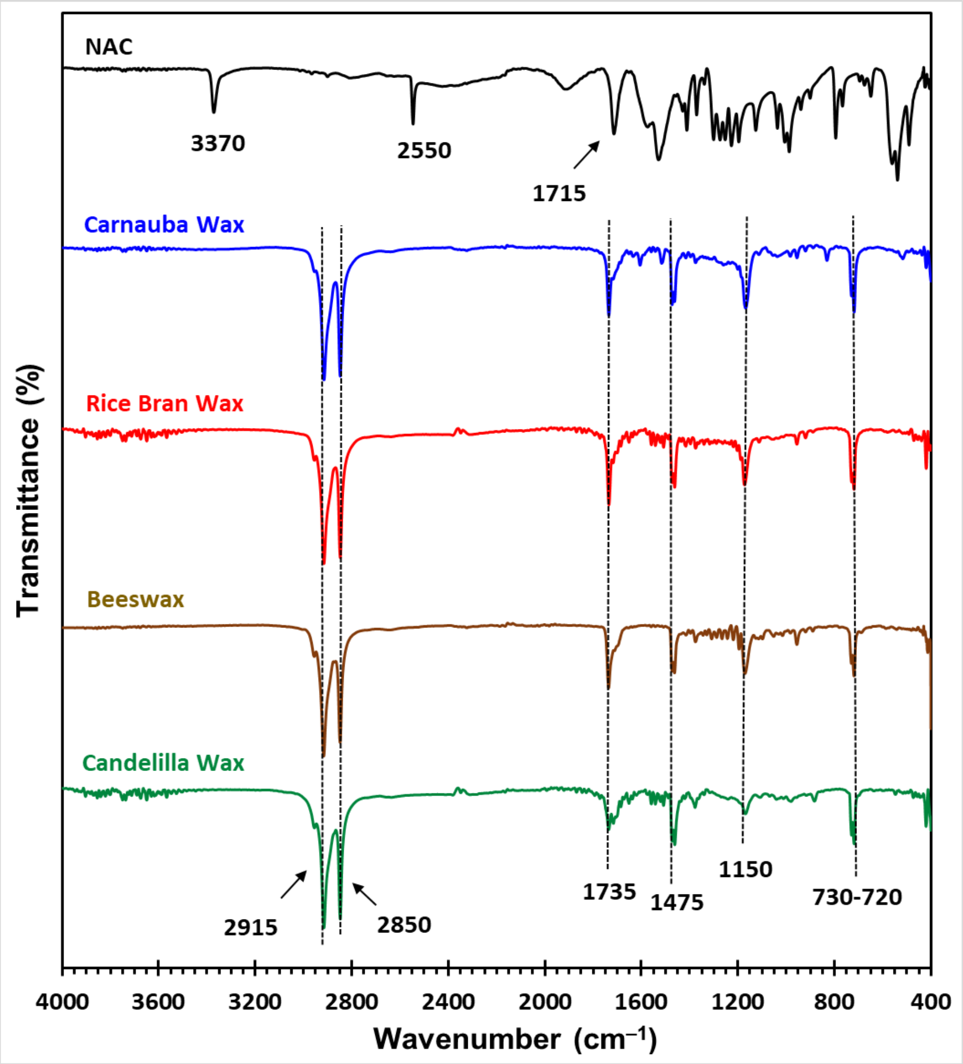

Figure S4. ATR-FT-IR spectra of NAC and pure hydrophobic coating materials (natural waxes).

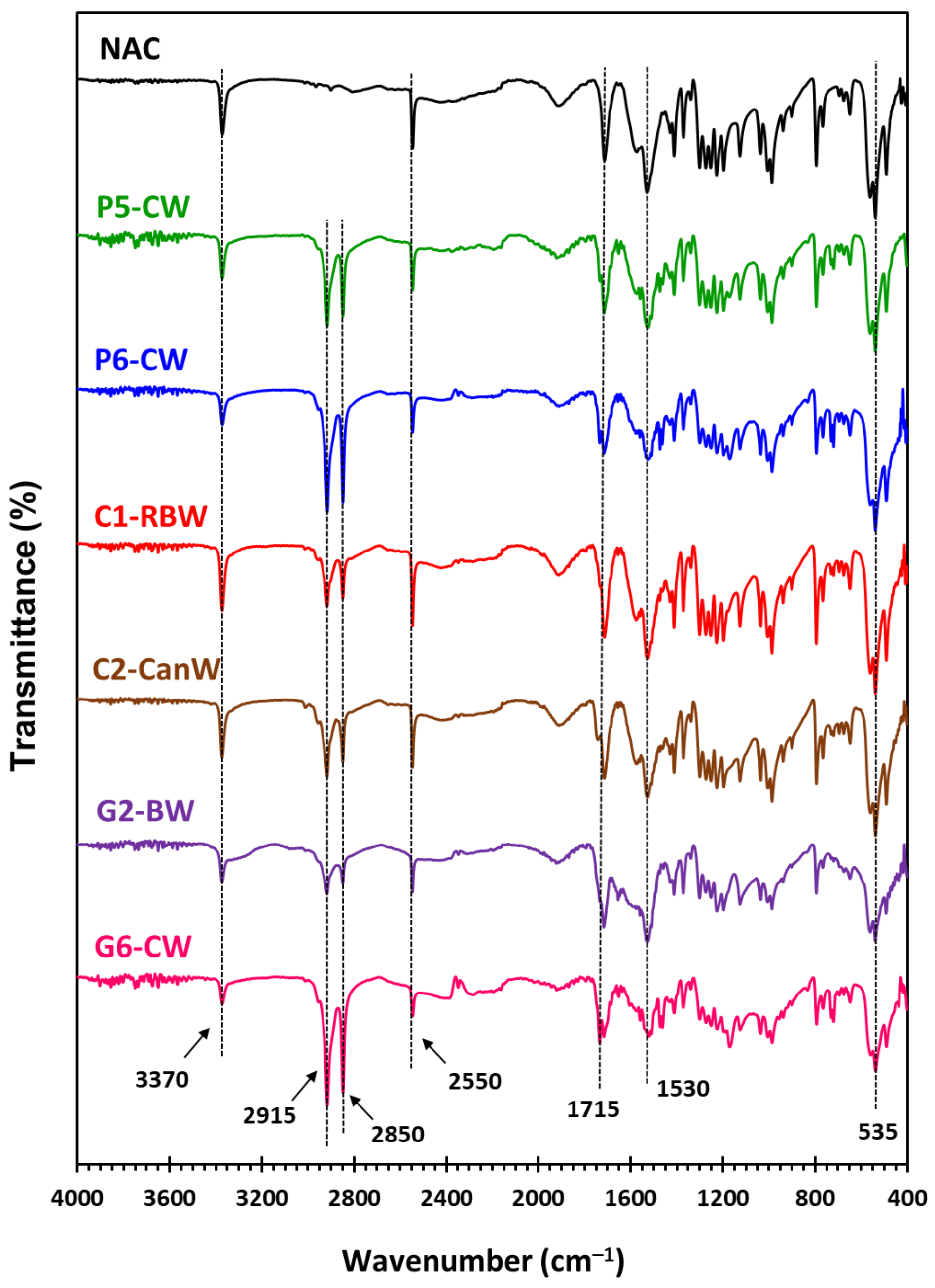

Figure S5. ATR-FTIR spectra of NAC and wax-coated samples.

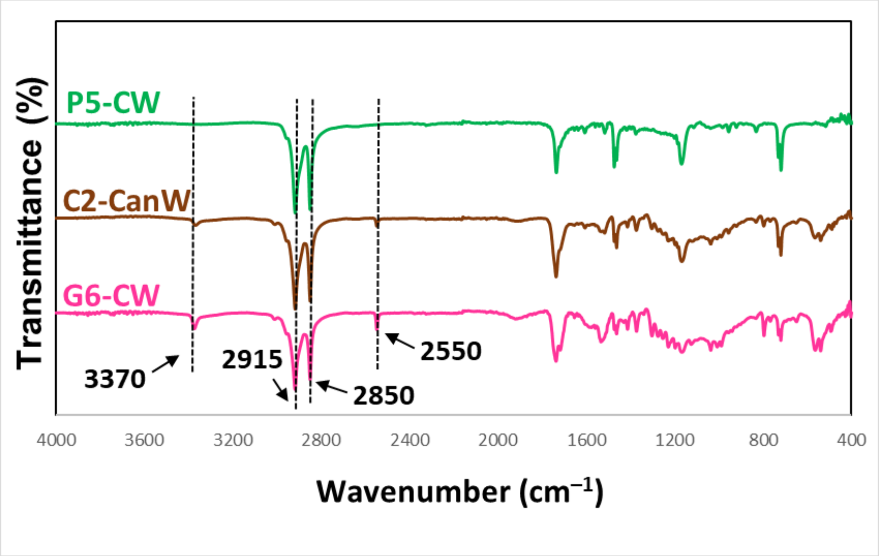

Figure S6. ATR-FTIR of the residual solid from three samples after exposing to water for 10 minutes.

The FTIR stretches that confirm the presence of NAC are the N–H stretching vibration at 3370 cm^{-1}, a specific sharp peak at 2550 cm^{-1} which is related to the free S–H stretching, the peak of the carbonyl group at 1715 cm^{-1}, the N–H bending at 1530 cm^{-1} [1], and another peak at 535 cm^{-1} related to stretching of carboxyl group.[1-3]

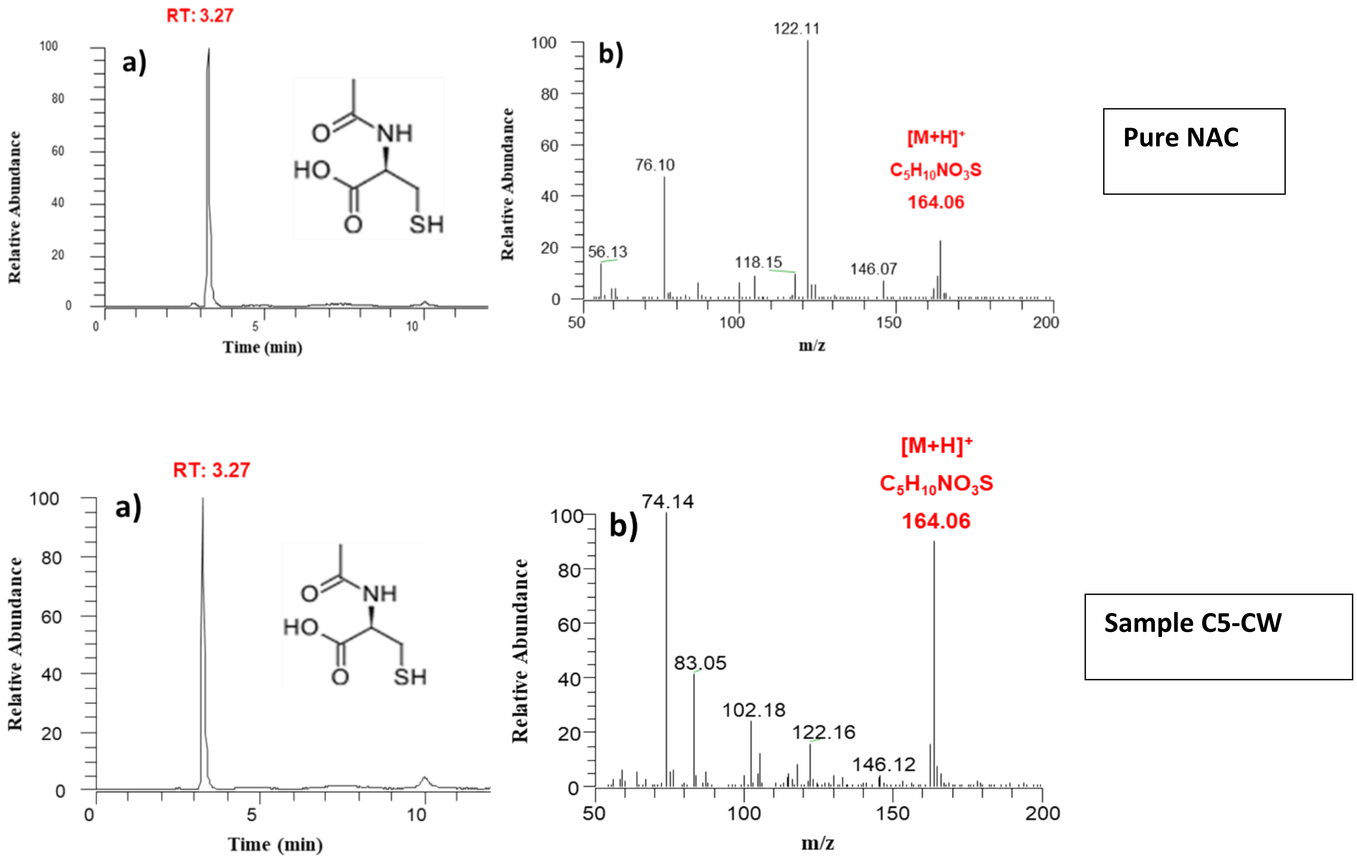

Figure S7. Top: a) Selected Reaction Monitoring (SRM) chromatogram of pure NAC at Retention time (RT) 3.27 min, b) mass spectrum of the pure NAC. Bottom: SRM chromatogram of C5-CW sample at the same RT, b) mass spectrum of the C5-CW sample by LC-MS.

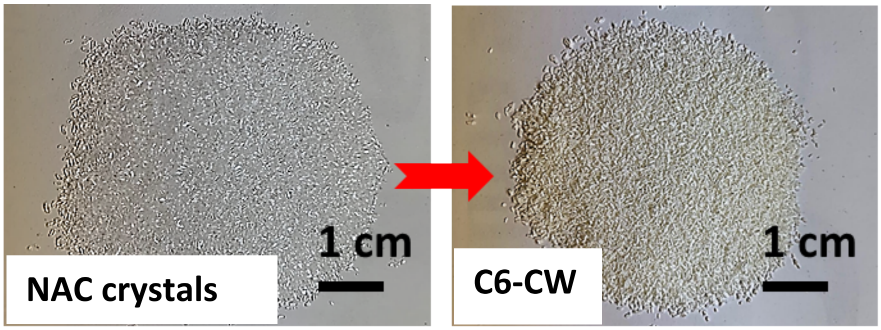

Figure S8. Photo of the NAC crystal before and after coating with CW.

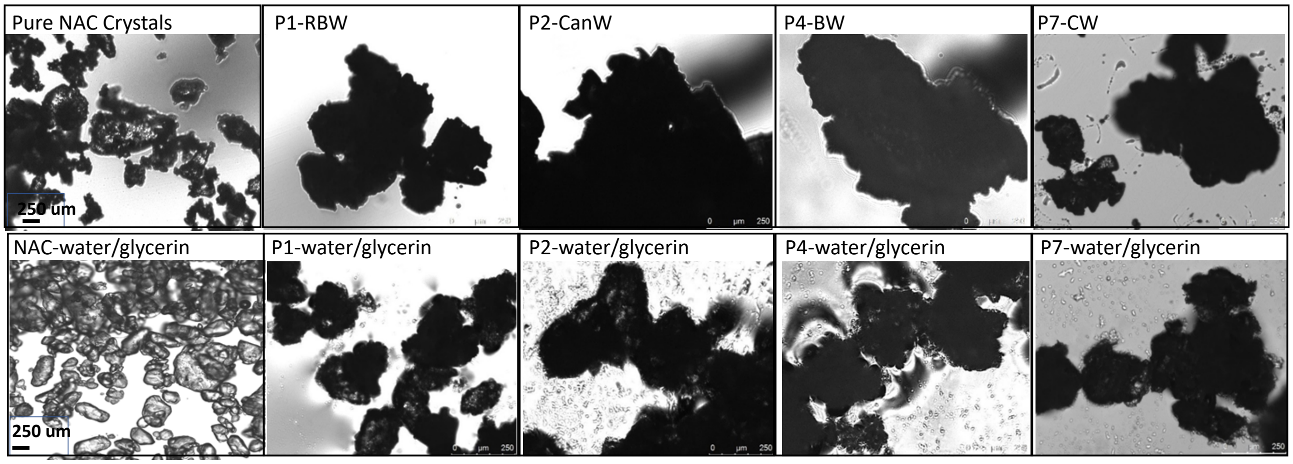

Figure S9. Microscopic images of the NAC Powder, 80 mesh, (P) coating with natural waxes (left bottom: microscopic image of pure NAC 80 mesh).

NAC powder contains very small NAC crystals (80 mesh). After coating, the small particles tend to stick to each other, therefore, the light microscope was not able to clearly show the particles.

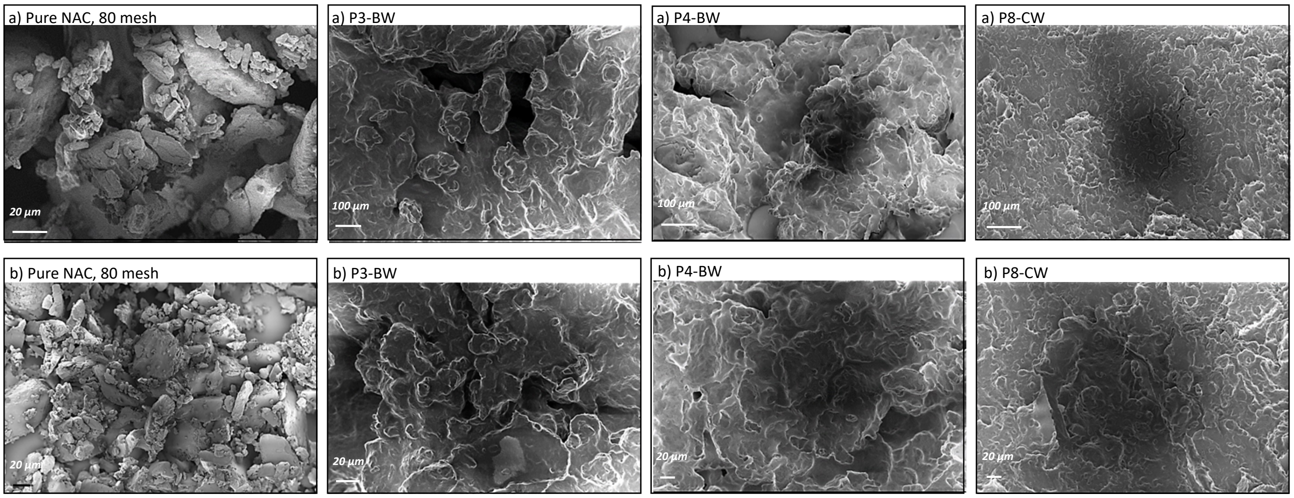

Figure S10. Left: SEM images of pure NAC powder, 80 mesh, (P); SEM of NAC powder coating with different waxes.

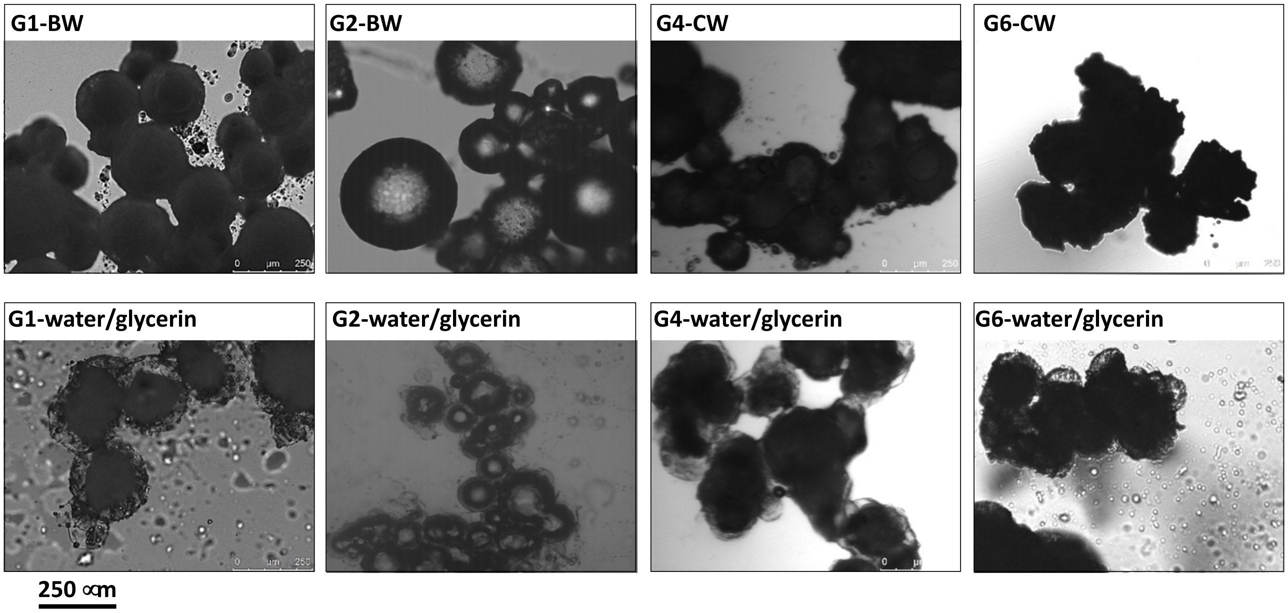

Figure S11: Microscopic images of the granules NAC (prepared via phase separation) coating with different waxes. The particles appear dark because of the wax coating and light does not penetrate through the particles.

1. D. L. Pavia, G. M. Lampman, G. S. Kriz and J. A. Vyvyan, *Introduction to spectroscopy*, Cengage Learning, 2008.
2. Z. Du, J. Liu, J. Zhai, H. Huang, S. Wei, T. Zhang, L. Ding and B. Liu, *Food hydrocolloids*, 2019, **96**, 377-384.
3. S. Pathan, P. Solanki and A. Patel, *Journal of Porous Materials*, 2017, **24**, 1105-1115.
